# National Diet and Nutrition Survey data reveal a decline in folate status in the United Kingdom population between 2008 and 2019

**DOI:** 10.1016/j.ajcnut.2023.10.006

**Published:** 2023-10-14

**Authors:** Kerry S. Jones, David Collins, Sarah R. Meadows, Albert Koulman, Polly Page

**Affiliations:** 1 Nutritional Biomarker Laboratory, MRC Epidemiology Unit, University of Cambridge, Cambridge, United Kingdom; 2 Nutrition Measurement Platform, MRC Epidemiology Unit, University of Cambridge, Cambridge, United Kingdom

**Keywords:** folic acid, neural tube defects, nutritional status, nutrition surveys, females of reproductive age

## Abstract

**Background:**

Folate is essential for healthy growth and development. Fortification of foods with folic acid can improve folate status and reduce risk of neural tube defects (NTD). Following concern around folate status in the United Kingdom, the United Kingdom government announced in 2021 the intention to introduce mandatory folic acid fortification.

**Objective:**

This study aimed to describe folate status in the United Kingdom population prior to the implementation of mandatory folic acid fortification of non-whole wheat (non-wholemeal) flour and to assess trends in folate status, including in females of reproductive age (FRA).

**Methods:**

Data were from the United Kingdom National Diet and Nutrition Survey Rolling Program (2008–2019), a cross-sectional, nationally representative survey of children and adults aged 1.5+ (*n* = 5792 with folate result). Serum folate concentration was measured by liquid chromatography tandem mass spectrometry (LC-MS/MS) and red blood cell (RBC) folate concentration by microbiological assay. Concentration data were compared against method-specific cut-offs and thresholds, and relationships were explored against demographic and lifestyle characteristics.

**Results:**

RBC and serum folate concentration significantly decreased by ∼3 percentage points per year between 2008 and 2019 in all age/sex groups. Prevalence of deficiency (RBC folate < 305 nmol/L) was highest in children aged 11 to 18 y (17% in 2016–2019). The proportion of FRA below the cut-off for increased risk of NTD (RBC folate < 748 nmol/L) increased from 69% to 89% between 2008 and 2019. Ethnicity, smoking status, and income were significant determinants of RBC and serum folate concentrations.

**Conclusions:**

These data reveal a decline in population folate status in the United Kingdom between 2008 and 2019 and a high prevalence of folate deficiency. A high proportion of FRA had RBC folate concentrations below the cut-off for increased risk of NTD. These data provide information on folate status in a population not currently exposed to mandatory folic acid fortification and are essential to model and assess its impact.

## Introduction

Folate is essential for DNA synthesis and cell growth, and folate requirements are generally higher during periods of rapid growth and development. Deficiency most commonly manifests as megaloblastic anemia due to the high demand for folate associated with the production of red blood cells (RBC). In pregnancy, folate deficiency can lead to neural tube defects (NTD), most frequently spina bifida and anencephaly. Low folate status is also associated with increased risk of cardiovascular disease, cancer, and neurological conditions [[1], [2], [3]].

Data from the surveillance of congenital anomalies in Europe (EUROCAT) suggest a prevalence of pregnancies affected by nongenetic NTD across regions of England and Wales between 2008 and 2020 of 12.4 (12.0–12.7) per 10,000 pregnancies, and 2.4 (2.3–2.6) per 10,000 live births [4]. Increased folic acid intake, through supplementation or food fortification, can increase folate status and reduce risk of NTD [2]. Fortification of wheat or maize flour is a cost-effective and efficacious method to increase folate intake in at-risk populations [2,5]. Mandatory fortification has been demonstrated to reduce the prevalence of NTD [6,7], and by 2022, folic acid fortification was mandated in 69 countries worldwide [8]. However, in Europe, as of May 2023, Kosovo and Moldova were the only countries to have implemented mandatory folic acid fortification of flour [8]. In the United Kingdom, fortification of foods with folic acid is permitted on a voluntary basis. In September 2021, the United Kingdom government announced its intention to implement the mandatory fortification of non-whole wheat (non-wholemeal) flour with folic acid [9]. At the time of writing, no precise date had been set for laying the legislation to amend the Bread and Flour Regulations 1998, but it is anticipated to be in 2024 [10].

Monitoring of population folate status is essential to provide an evidence base for the introduction, implementation, and evaluation of folic acid fortification. A systematic review from 2018 reported that 38 countries had nationally representative survey data on folate status in females of reproductive age (FRA) assessed with RBC or serum folate [11]. Although data from some countries that have introduced folic acid fortification have demonstrated improvements in folate status [12], few countries have nationally representative data on both pre- and postfortification. In addition to the dearth of data on population folate status globally, the use of different assays and related cut-offs for deficiency/sufficiency confound the interpretation of collated data [1,13].

The World Health Organization (WHO) recommends the use of the microbiological assay (MBA) to assess population RBC folate concentration in relation to NTD risk and has set population cut-offs accordingly [13,14]. Serum or plasma folate concentration is a shorter-term biomarker of folate status and does not have an associated cut-off for the prevention of NTD, but it does provide information on dietary exposure to folic acid [1]. Measurement by liquid chromatography mass spectrometry (LC-MS/MS) of the different serum forms of folate is relevant to studying the impact of fortification and potential impact of free folic acid in relation to health outcomes [1].

The United Kingdom National Diet and Nutrition Survey Rolling Program (NDNS RP) is a cross-sectional annual survey of the United Kingdom general population and has collected data on micronutrient status, including RBC folate by MBA and serum folate by LC-MS/MS since 2008 [15]. Here, we describe folate status in the United Kingdom population. We present data on folate status prior to the mandatory implementation of folic acid fortification and assess trends in folate status, including in FRA, between 2008 and 2019.

## Methods

The NDNS RP is a United Kingdom government-funded, population-representative, cross-sectional survey of dietary and nutritional intake and status. Details of the survey program, including its history, funding, organization, and available data, were published recently [15]. Survey results and reports are also available [16]. In summary, dietary and other questionnaire data were collected annually from ∼500 adults (≥19 y) and 500 children (aged 1.5 to 18) living in the United Kingdom (England, Wales, Scotland, and Northern Ireland). Around 500 of these participants each year also provided a blood sample for nutritional biomarker analysis. A summary of the sampling design for the survey is available [15], and further details are included in the reports and appendixes for each survey cycle [16].

NDNS RP conforms to the Declaration of Helsinki and operates under United Kingdom National Health Service Health Research Authority Research Ethics Committee approval (Years 1–5, Oxfordshire REC A, REF 07/H0604/113; Years 6–10 and 11–15, East of England-Cambridgeshire South REC, REF 13/EE/0016).

Here, we report on NDNS RP data from 2008 to 2019 organized by survey year: Years 1–2, 2008/09–2009/10; Years 3–4, 2010/11–2011/2012; Years 5–6, 2012/13–2013/14; Years 7–8, 2014/15–2015/16; Years 9–11, 2016/17–2018/19.

### Sample collection

Venous whole blood (WB) samples were collected by venipuncture from overnight fasted participants (with the exception of children aged 1.5 to 3 and participants who were unable or unwilling to fast). EDTA blood tubes were collected for the measurement of WB folate and hematocrit, and serum tubes for serum folate.

The EDTA WB sample was mailed at ambient temperature to the processing laboratory (Addenbrooke’s Hospital NDNS RP Years 1 to 10; MRC Epidemiology Unit NDNS RP Year 11). A 100 μL aliquot of WB was removed and added to 1 mL of 1% ascorbic acid to preserve folate. Samples were subsequently stored frozen (-70°C or colder). A complete (full) blood count (Siemens Advia 2120) was performed on the remaining EDTA WB sample and provided the measure of hematocrit used to calculate RBC folate.

After collection, blood samples for the preparation of serum were transported chilled to a local laboratory for processing. Serum was stored frozen and subsequently sent to the central NDNS laboratory (MRC Elsie Widdowson Laboratory NDNS RP Years 1 to 10 and MRC Epidemiology Unit NDNS RP Years 11) on dry ice and stored at -70°C until analysis or onward transportation on dry ice to the analyzing laboratory.

### Whole blood folate

Samples for WB folate measurement were sent to the Centers for Disease Control and Prevention (CDC) on dry ice. WB folate was measured using the MBA with 5-methyltetrahydrofolate (5-MTHF) calibrator [1,17,18]. This assay is based on the folate-dependent growth of *Lactobacillus rhamnosus* and is the recommended assay to assess RBC folate [1,14]. Quality control (QC) was monitored with 4 WB QC pools. Accuracy was assessed by periodic assaying of the 1^st^ WHO International Standard for Whole Blood Folate 95/528 (NIBSC, Hertfordshire, United Kingdom) and by successful participation in the United Kingdom NEQAS Hematinic program (United Kingdom NEQAS). Assay performance data can be obtained from the appendixes of the relevant NDNS RP reports [16,[19], [20], [21]].

### Serum folate

Serum folate was assessed by the measurement of folate vitamers in serum by LC-MS/MS and was performed at the CDC for NDNS RP Years 1–6 [22] and at the central NDNS laboratory for NDNS RP Years 7–11 [23]. The NDNS laboratory method was adapted from the CDC method. The method transition between laboratories was performed in collaboration with CDC and included incurred sample reanalysis of 250 samples from NDNS RP Year 4 (Supplemental Figure 1) and was approved at the time by the NDNS Project Board (October 2015). Participation in an interlaboratory comparison study with the CDC in 2015 (conducted during analysis of NDNS samples from Years 7 and 8) demonstrated comparable agreement, precision, and accuracy between the CDC and the NDNS central laboratory (laboratory: Group 2_14 in the manuscript) and accuracy within 3% of NIST Standard Reference Material targets [24]. The LC-MS/MS method measured 6 folate vitamers (5-MTHF, folic acid, tetrahydrofolate (THF), 5-formyltetrahydrofolate (5-FTHF), 5,10 methenyltetrahydrofolate (CH+THF), and pyrazino-s-triazine derivative of 4α-hydroxy-5-methyltetrahydrofolate (MeFox), which were summed to provide the serum total folate concentration. Where results were below the limit of quantitation (LOQ) or detection (LOD) of the instrument, values were imputed using the LOQ or LOD divided by the square root of 2 [25,26] and used to calculate serum total folate. Because MeFox is an oxidation product of 5-MTHF, there is a suggestion that it should not be included in serum total folate [27], and we therefore report here serum total folate both with and without MeFox. At the central NDNS laboratory, internal QC for serum folate measurements was performed with 2 inhouse serum controls and a serum pool spiked with exogenous 5-FTHF, THF, and CH+THF. Accuracy was monitored by regular measurement of NIST Standard Reference Material 1950 (National Institute of Standards and Technology, MD, USA) (Supplemental Table 1). Quality assurance was provided by participation in United Kingdom NEQAS and CDC VITAL-EQA schemes. Additional assay performance data can be obtained from the appendices of the relevant NDNS RP reports [16,[19], [20], [21]].

### Calculation of RBC folate

Where a result was obtained for WB folate, RBC folate was calculated according to the equation:

RBC folate = {WB folate–[serum folate x (1–hematocrit)]}/hematocrit

Where serum folate or hematocrit values were unavailable, surrogate values of 18 nmol/L and 0.4 L/L were used, respectively. If neither value was available, then no result was calculated. Supplemental Table 2 provides frequencies for the use of surrogate values.

### Thresholds and deficiency cut-offs

Prevalence of folate deficiency or insufficiency was based on established, method-appropriate cut-offs. For the results of the MBA, the RBC folate cut-off of 305 nmol/L represents folate deficiency associated with increased risk of megaloblastic anemia. For FRA, the threshold of 748 nmol/L represents the level below which there is an increased risk of NTD [28]. Serum folate cut-offs of < 7 nmol/L and < 13 nmol/L represent deficiency and possible deficiency in relation to risk of megaloblastic anemia, respectively [29].

### Statistical methods

Due to the skewness of the data, all results are presented as either geometric mean or median with 2.5 and 97.5 percentiles. The results tables are primarily descriptive. All data were weighted for sample selection and participant response and are, therefore, representative of the United Kingdom population. The weights were adjusted for selection across addresses, households, and individuals and nonresponse to a nurse’s visit and collection of blood sample. Further details are published in the NDNS RP reports [16].

The proportion of ambient temperature mailed WB EDTA samples for RBC folate analysis received by the processing laboratory > 48 h after collection has increased over the course of the survey since 2008, from ∼10% in Years 6–9 to 30% in Years 10–11. Due to the possible instability of folate at ambient temperature [1] and temporal increases in the number of samples received >48 h after collection, from Year 10 onwards, samples received > 48 h after collection were excluded from the data analysis.

Time trend analysis was performed using linear regression, with each survey year divided into quarters to fully characterize the trends over time for each age group. The slope of the regression line represents the average year-by-year change.

Multiple linear regression was used to investigate associations between folate concentration and status and participant and demographic characteristics. Characteristics were self-reported with the exception of height and weight used to calculate body mass index (BMI), which were measured during the fieldworker visit. Statistical models included all variables described below, as well as NDNS RP survey year. Natural log values of the outcome measures were included, and ratios of geometric means between groups were used to calculate the percentage difference between categorical variables. Included variables were age at the time of blood collection, sex, BMI, ethnic group (self-defined and categorized as either white, mixed, black, Asian, or other), country of residence (England, Scotland, Wales, or Northern Ireland), whether a current smoker and a single question on supplement use (Did you take any vitamins, minerals or other food supplements today? Yes or No). Due to the low number of young participants with folate concentrations below the cut-offs, regression analysis for these associations was only performed with participants aged ≥ 11.

In NDNS RP reports, BMI cut-offs are reported differently for adults and children: in adults, the established WHO cut-offs for underweight, normal weight, overweight, obese, and severely obese are reported, and in children, the cut-offs for normal, overweight, and obese are based on population-specific centiles. To combine adult and children's BMI data in the regression analyses, the adult BMI categories were collapsed into 3 categories, with underweight participants (*n* = 37) included in the normal category and severely obese (*n* = 119) in the obese category. Household income is described as low, middle, or high and is categorized as equivalized income, i.e., adjusted household income for different demands on resources considering the household size and composition [30].

## Results

For RBC folate and serum folate, the total sample sizes for all years (2008 to 2019) by age group were: children aged 1.5 to 3: 122 and 118; children aged 4 to 10: 557 and 569; children aged 11 to 18: 1053 and 1,106; adults aged 19 to 64: 2856 and 3104; and adults 65+ y: 807 and 891, respectively. A participant flow chart is provided in Supplemental Figure 2, and sample sizes for RBC and serum folate in age and sex groups are presented in Supplemental Table 3. A table of participant characteristics is included in Supplemental Table 4.

Geometric mean RBC folate (Table 1) and serum folate (Table 2) concentrations were generally higher in the youngest (4–10 y) and oldest age groups (65+ y) and lowest in children (11–18 y) and decreased between 2008 and 2019.

**TABLE 1:** Red blood cell (RBC) folate concentration by age and sex groups in the NDNS RP 2008 – 2019^1^

Age	Sex	RBC folate, nmol/L (geometric mean (2.5^th^, 97.5^th^ percentiles)) 2				
		NDNS RP years				
		1 – 2	3 – 4	5 – 6	7 – 8	9 – 11
1.5 – 3 y	All	-	-	710 (279, 1354)	678 (342, 1074)	-
4 – 10 y	All	663 (320, 1204)	662 (334, 1466)	634 (299, 1211)	537 (248, 961)	544 (289, 1084)
	Male	741 (309, 1216)	669 (379, 1267)	677 (248, 1159)	560 (249, 882)	574 (199, 1140)
	Female	609 (320, 1030)	654 (260, 1669)	596 (323, 1138)	514 (186, 947)	511 (302, 814)
11 – 18 y	All	517 (250, 1060)	488 (202, 1070)	485 (219, 1052)	416 (183, 831)	408 (169, 855)
	Male	554 (305, 1079)	533 (260, 1070)	490 (187, 937)	456 (237, 823)	434 (172, 999)
	Female	478 (231, 868)	450 (141, 997)	481 (211, 1049)	377 (165, 824)	380 (142, 677)
19 – 64 y	All	606 (282, 1283)	574 (258, 1366)	539 (266, 1269)	508 (206, 1197)	489 (200, 1284)
	Male	608 (332, 1220)	561 (231, 1227)	542 (264, 1177)	531 (261, 1141)	498 (231, 1159)
	Female	604 (262, 1307)	587 (271, 1496)	536 (267, 1290)	485 (192, 1260)	480 (160, 1363)
65+ y	All	666 (293, 1815)	697 (308, 1518)	610 (262, 1752)	585 (242, 1813)	553 (213, 1413)
	Male	678 (272, 1926)	647 (289, 1397)	601 (156, 1754)	523 (239, 1078)	573 (202, 1335)
	Female	658 (281, 1767)	744 (307, 1531)	617 (266, 1428)	640 (233, 1921)	538 (212, 1387)
16 – 49 y	FRA	581 (258, 1278)	543 (255, 1452)	496 (234, 1085)	430 (191, 999)	447 (140, 1456)

Abbreviations: FRA, females of reproductive age; NDNS RP, National Diet and Nutrition Survey Rolling Program; RBC folate, red blood cell folate; NDNS RP Years are: Years 1–2, 2008/09–2009/10; Years 3–4, 2010/11–2011/2012; Years 5–6, 2012/13–2013/14; Years 7–8, 2014/15–2015/16; Years 9–11, 2016/17–2018/19

1 Where the group size is less than 30 participants, the results are not included

2 RBC folate concentration is calculated from whole blood folate concentration, serum folate concentration, and hematocrit (see Methods section for further details)

**TABLE 2:** Serum folate concentration by age and sex groups in NDNS RP 2008 – 2019^1^

Age	Sex	Serum folate, nmol/L (geometric mean (2.5^th^, 97.5^th^ percentiles)) 2				
		NDNS RP years				
		1 – 2	3 – 4	5 – 6	7 – 8	9 – 11
1.5 – 3 y	All	-	-	32.1 (10.0, 88.5)	-	-
4 – 10 y	All	26.2 (11.6, 65.5)	25.7 (8.1, 69.5)	25.6 (11.1, 57.1)	21.9 (8.2, 53.2)	20.9 (7.9, 46.4)
	Male	30.9 (10.5, 80.1)	25.6 (8.6, 67.7)	28.5 (13.9, 55.6)	21.3 (9.8, 53.3)	21.8 (6.7, 49.9)
	Female	23.5 (11.3, 44.4)	25.9 (7.2, 76.7)	22.8 (9.9, 52.7)	22.6 (7.9, 49.7)	20.0 (8.2, 41.2)
11 – 18 y	All	15.9 (6.2, 40.5)	15.0 (5.8, 46.3)	14.7 (5.3, 39.9)	11.6 (4.7, 28.7)	13.0 (5.8, 34.6)
	Male	15.7 (5.9, 39.7)	15.8 (5.8, 59.7)	14.9 (5.0, 39.3)	12.2 (4.7, 25.2)	13.2 (5.9, 34.6)
	Female	16.0 (6.9, 40.6)	14.4 (5.7, 32.9)	14.5 (5.9, 38.9)	10.8 (3.9, 30.7)	12.9 (5.5, 30.1)
19 – 64 y	All	17.6 (6.8, 48.8)	16.4 (6.2, 55.5)	15.5 (5.7, 54.4)	14.2 (5.4, 43.4)	14.0 (5.2, 57.3)
	Male	17.1 (6.8, 46.7)	15.1 (6.2, 39.1)	15.0 (5.8, 54.2)	13.9 (5.5, 33.3)	13.1 (5.2, 43.1)
	Female	18.0 (6.8, 48.7)	17.8 (6.4, 73.7)	16.1 (5.7, 54.1)	14.6 (5.2, 48.6)	14.9 (5.1, 65.6)
65+ y	All	20.4 (6.5, 69.1)	21.3 (8.1, 68.0)	17.7 (6.3, 53.1)	18.1 (5.6, 73.4)	17.4 (5.6, 66.6)
	Male	18.8 (5.3, 61.7)	19.3 (8.2, 54.0)	18.0 (6.1, 52.4)	14.9 (4.9, 40.1)	17.7 (5.6, 53.2)
	Female	21.5 (6.5, 69.3)	23.1 (7.7, 73.2)	17.5 (6.5, 51.4)	21.3 (5.4, 74.9)	17.2 (5.6, 68.1)
16 – 49 y	FRA	17.2 (7.0, 44.9)	16.7 (6.5, 64.1)	14.5 (4.7, 41.5)	12.7 (5.1, 43.7)	14.0 (4.7, 65.1)

Abbreviations: FRA, females of reproductive age; NDNS RP, National Diet and Nutrition Survey Rolling Program. NDNS RP Years are: Years 1–2, 2008/09–2009/10; Years 3–4, 2010/11–2011/2012; Years 5–6, 2012/13–2013/14; Years 7–8, 2014/15–2015/16; Years 9–11, 2016/17–2018/19

1 Where the group size is less than 30 participants, the results are not included.

2 Serum folate is the sum of 6 folate vitamer concentrations (5-methyltetrahydrofolate (5-MTHF), folic acid, tetrahydrofolate (THF), 5-formyltetrahydrofolate (5-FTHF), 5,10 methenyltetrahydrofolate (CH+THF) and pyrazino-s-triazine derivative of 4α-hydroxy-5-methyltetrahydrofolate (MeFox).

Exclusion of MeFox concentration reduced serum folate concentration by around 1 nmol/L across all groups (Supplemental Table 5). However, patterns across time and differences between groups were similar to those with serum folate concentration calculated with MeFox. Median folic acid concentrations in all groups ranged between 0.07 and 0.47 nmol/L (Table 3). For folic acid, due to the number of samples below the sensitivity limits of the analytical method, the 2.5 percentile is largely determined by the LOD. Variation in this 2.5 percentile between NDNS years reflects differences and changes in instrument sensitivity between laboratories and over time. Across all survey years, 33% of participants had a folic acid result below the LOD.

**TABLE 3:** Serum folic acid concentration by age and sex groups in NDNS RP 2008 – 2019^1^

Age	Sex	Serum folic acid, nmol/L (median (2.5^th^, 97.5^th^ percentiles))				
		NDNS RP years				
		1 – 2	3 – 4	5 – 6	7 – 8	9 – 11
1.5 – 3 y	All	-	-	0.47 (0.10, 3.85)	-	-
4 – 10 y	All	0.43 (0.06, 1.04)	0.30 (0.14, 0.86)	0.39 (0.10, 1.12)	0.25 (0.04, 0.93)	0.13 (0.04, 0.63)
	Male	0.45 (0.06, 1.07)	0.26 (0.14, 0.87)	0.39 (0.10, 1.13)	0.25 (0.04, 0.61)	0.14 (0.04, 0.57)
	Female	0.42 (0.06, 0.76)	0.36 (0.14, 0.77)	0.30 (0.10, 1.12)	0.24 (0.04, 1.06)	0.13 (0.04, 0.66)
11 – 18 y	All	0.33 (0.06, 1.04)	0.27 (0.14, 0.81)	0.25 (0.10, 0.80)	0.13 (0.04, 0.57)	0.11 (0.04, 0.55)
	Male	0.33 (0.06, 0.97)	0.26 (0.14, 1.06)	0.24 (0.10, 0.87)	0.18 (0.04, 0.64)	0.07 (0.04, 0.55)
	Female	0.35 (0.06, 1.01)	0.28 (0.14, 0.80)	0.25 (0.10, 0.73)	0.07 (0.04, 0.43)	0.13 (0.04, 0.48)
19 – 64 y	All	0.39 (0.06, 1.14)	0.26 (0.14, 1.06)	0.23 (0.10, 1.15)	0.15 (0.04, 0.79)	0.11 (0.04, 0.90)
	Male	0.37 (0.06, 1.15)	0.27 (0.14, 0.91)	0.22 (0.10, 1.13)	0.15 (0.04, 0.72)	0.07 (0.04, 0.66)
	Female	0.41 (0.06, 1.06)	0.24 (0.14, 1.17)	0.23 (0.10, 1.13)	0.13 (0.04, 0.92)	0.11 (0.04, 1.01)
65+ y	All	0.35 (0.06, 1.21)	0.40 (0.14, 1.39)	0.22 (0.10, 1.37)	0.14 (0.04, 1.10)	0.14 (0.04, 1.34)
	Male	0.27 (0.06, 0.86)	0.26 (0.14, 1.36)	0.29 (0.10, 1.76)	0.13 (0.04, 0.92)	0.12 (0.04, 1.04)
	Female	0.36 (0.06, 1.58)	0.45 (0.14, 1.31)	0.16 (0.10, 0.98)	0.15 (0.04, 16.15)	0.15 (0.04, 1.34)
16 – 49 y	FRA	0.38 (0.06, 1.14)	0.25 (0.14, 1.05)	0.22 (0.10, 1.08)	0.10 (0.04, 0.89)	0.11 (0.04, 1.00)

Abbreviations: FRA, females of reproductive age; NDNS RP, National Diet and Nutrition Survey Rolling Program. NDNS RP Years are: Years 1–2, 2008/09–2009/10; Years 3–4, 2010/11–2011/2012; Years 5–6, 2012/13–2013/14; Years 7–8, 2014/15–2015/16; Years 9–11, 2016/17–2018/19

1 Where the group size is less than 30 participants, the results are not included.

**FIGURE 1:**
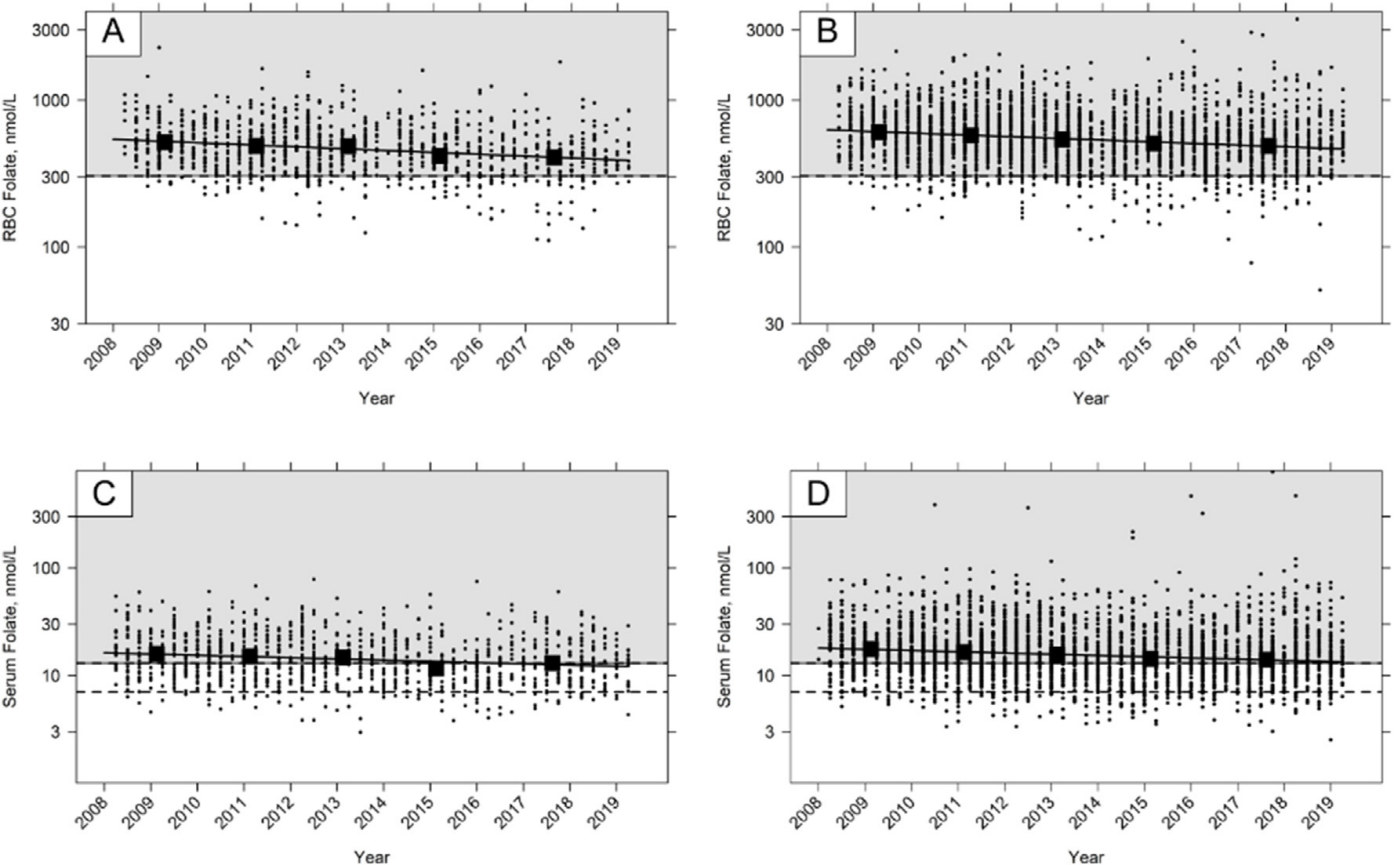
Time trend in red blood cell (RBC) and serum folate concentration in the NDNS RP. Figure 1A, RBC folate in children aged 11–18; Figure 1B, RBC folate in adults aged 19–64; Figure 1C, Serum folate in children aged 11–18; Figure 1D, Serum folate in adults aged 19–64. Data points are individual participant concentrations on the log scale. Dashed lines indicate cut-offs for deficiency (RBC folate <305 nmol/L; serum folate <7 nmol/L) or possible deficiency (serum folate <13 nmol/L), and the background color indicates the concentrations above (gray) or below (white) the cut-offs. Time trend analysis was performed using linear regression, with each survey year divided into quarters to fully characterize the trends over time for each age group. The slope of the regression line represents the average year-by-year change. Solid closed symbols indicate the geometric mean concentration for the reporting periods (see text). See Supplemental Table 2 for sample sizes.

Time trend analysis confirmed temporal trends in folate status (Figure 1; Supplemental Table 6). Geometric mean RBC folate concentration significantly decreased by an average of 2 to 3% per year in all age and sex groups (Supplemental Table 6), equivalent to a 22 to 31% decrease in RBC folate concentration between 2008 and 2019. A similar pattern was observed for serum folate (Figure 1; Supplemental Table 6) that decreased by an average of between 2 and 4% per year in all groups, a 16 and 34% decrease over the 11-year period.

**FIGURE 2:**
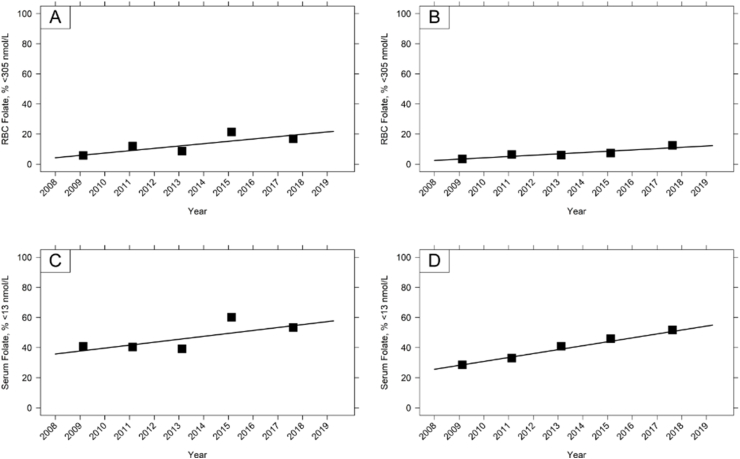
Time trend in the prevalence of folate deficiency in NDNS RP. Figure 2A, RBC folate deficiency (<305 nmol/L) in children aged 11–18; Figure 2B, RBC folate deficiency (<305 nmol/L) in adults aged 19–64; Figure 2C, Serum folate possible deficiency (<13 nmol/L) in children aged 11–18; Figure 2D, Serum folate possible deficiency (<13 nmol/L) in adults aged 19–64. Time trend analysis was performed using linear regression, with each survey year divided into quarters to fully characterize the trends over time for each age group. The slope of the regression line represents the average year-by-year change. Solid closed symbols indicate the mean prevalence of deficiency for the reporting periods. See Supplemental Table 3 for sample sizes.

Based on the cut-offs for deficiency for megaloblastic anemia, deficiency was higher in children aged 11 to 18 y than in other age groups (Table 4); in the most recent survey cycle, 17% had an RBC folate concentration <305 nmol/L whereas in adults (19–64 y), and older adults (65+ y) concentrations were 13% and 11%, respectively. The observed changes in RBC folate concentration over time were associated with significant increases in the prevalence of folate deficiency (Figure 2; Supplemental Table 6). Similarly, the prevalence of possible deficiency (serum folate <13 nmol/L) significantly increased by an average of 1 to 3 percentage points in most sex and age groups between 2008 and 2019, including both in children (4–10 y, 11–18 y) and adults (19–64 y) (Figure 2; Supplemental Table 6), where increases were equivalent to 16, 21 and 29 percentage point increases over the whole 11-y period, respectively.

**TABLE 4:** Prevalence of folate concentration less than cut-offs by age and sex groups in NDNS RP 2008 – 2019^1^

Age	Sex	RBC folate					Serum folate									
		% <305 nmol/L^2^					% <7 nmol/L^2^					<13 nmol/L^2^				
		NDNS RP Years					NDNS RP Years									
		1 – 2	3 – 4	5 – 6	7 – 8	9 – 11	1 – 2	3 – 4	5 – 6	7 – 8	9 – 11	1 – 2	3 – 4	5 – 6	7 – 8	9 – 11
1.5 – 3 y	All	-	-	2	0	-	-	-	0	-	-	-	-	3	-	-
4 – 10 y	All	1	1	3	7	4	0	0	0	0	1	3	8	5	16	17
	Male	2	0	4	4	5	0	0	0	0	1	4	7	0	12	15
	Female	0	2	1	9	1	0	0	0	0	0	3	10	9	20	19
11 – 18 y	All	6	12	9	21	17	3	4	5	10	9	41	40	39	60	53
	Male	2	8	9	15	16	5	5	8	9	11	44	43	34	49	55
	Female	10	15	8	28	18	2	3	3	12	6	38	38	44	73	52
19 – 64 y	All	4	6	6	7	13	3	4	7	10	11	29	33	41	46	52
	Male	0	9	3	3	10	3	5	7	8	10	28	37	40	46	58
	Female	6	4	9	11	15	3	4	6	11	11	29	29	42	45	46
65+ y	All	4	1	8	12	11	3	0	5	6	7	28	25	27	32	34
	Male	0	3	10	14	9	2	0	8	6	9	33	33	24	38	31
	Female	7	0	7	10	13	3	0	3	6	5	24	19	29	27	36
		RBC folate % <748 nmol/L^2^														
16 – 49 y	FRA	69	81	84	91	89	2	3	7	15	13	33	33	46	57	52

Abbreviations: FRA, females of reproductive age; NDNS RP, National Diet and Nutrition Survey Rolling Program; RBC folate, red blood cell folate. NDNS RP Years are: Years 1–2, 2008/09–2009/10; Years 3–4, 2010/11–2011/2012; Years 5–6, 2012/13–2013/14; Years 7–8, 2014/15–2015/16; Years 9–11, 2016/17–2018/19

1 Where the group size is less than 30 participants the results are not included.

2 For definitions of cut-offs see Methods section of the text.

**FIGURE 3:**
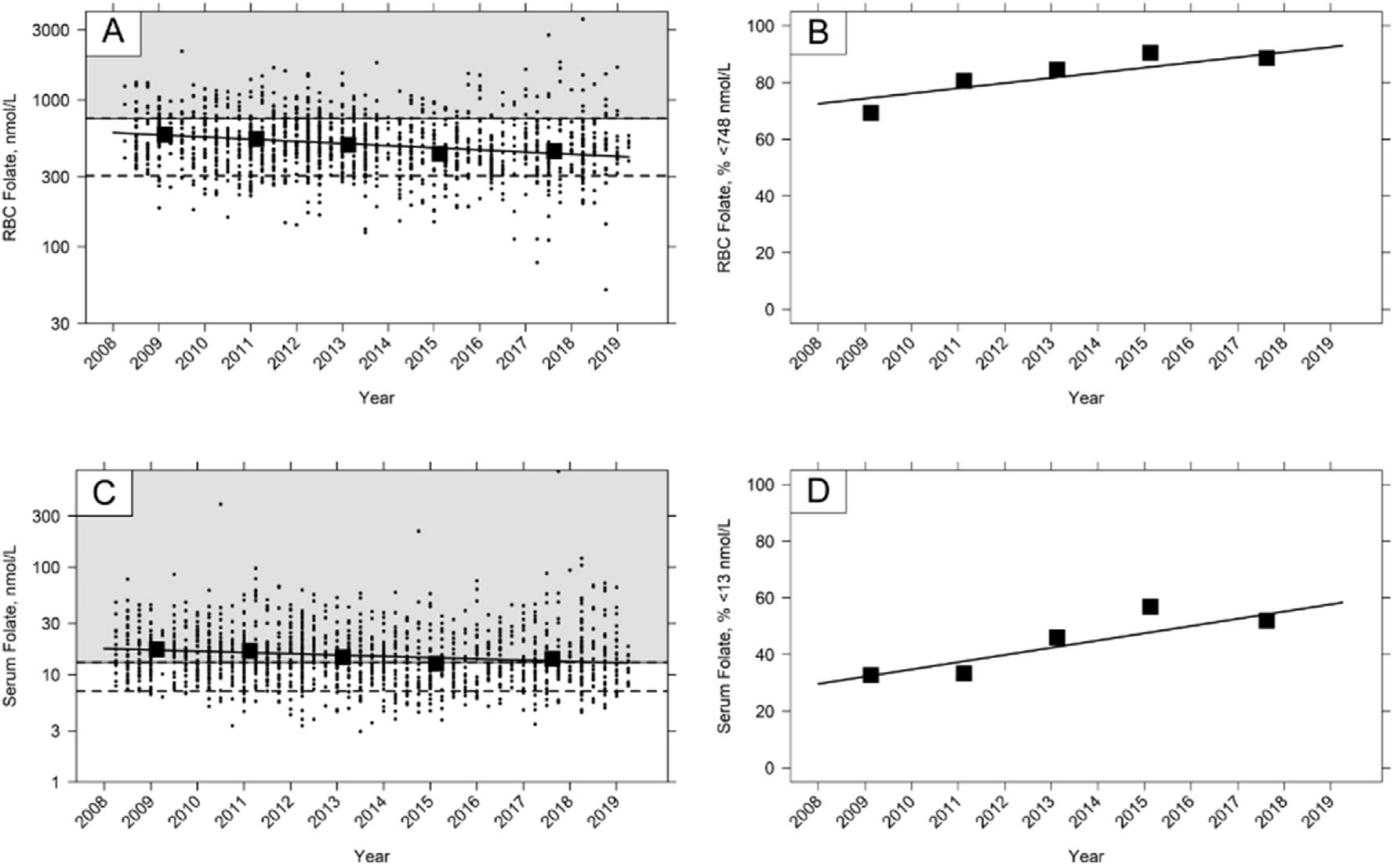
Red blood cell (RBC) and serum folate concentration and prevalence are less than selected cut-offs in females of reproductive age (FRA) in the NDNS RP. Figure 3A, RBC folate concentration; Figure 3B, Prevalence of RBC folate concentration <748 nmol/L; Figure 3C, Serum folate concentration; Figure 3D, Prevalence of serum folate possible deficiency (<13 nmol/L). Figures 3A and 3C: Data points are individual participant concentrations on the log scale. Dashed lines indicate cut-offs for deficiency (RBC folate <305 nmol/L; <748 nmol/L, or serum folate <7 nmol/L) or possible deficiency (serum folate <13 nmol/L), and the background color indicates concentrations above (gray) or below (white) a selected cut-off. All figures: Time trend analysis was performed using linear regression with each survey year divided into quarters to fully characterize the trends over time for each age group. The slope of the regression line represents the average year-by-year change. Solid closed symbols indicate the geometric mean concentration or mean prevalence rate for the reporting periods. See Supplemental Table 2 for sample sizes.

Data were analyzed for FRA separately (16–49 y) and, as for adults, RBC folate (Table 1; Figure 3) and serum folate (Table 2; Figure 3) concentrations decreased between 2008 and 2019 (Figure 3; Supplemental Table 6). The prevalence of FRA with RBC folate <748 nmol/L increased from 69 to 89 percentage points (Table 4), a significant 2 (CI 1, 3) percentage points per year increase.

Regression analysis showed that RBC and serum folate concentrations were significantly lower (13% and 7%, respectively) in older children (11–18 y) and higher (10% and 17%, respectively) in older people (65+ y) compared with adults aged 19–64 (Table 5). RBC and serum folate concentrations were significantly lower in the black ethnic grouping compared with the white ethnic grouping (23% and 16%, respectively). Smokers had significantly lower and supplement users significantly higher RBC and serum folate concentrations compared to nonsmokers and nonsupplement users, respectively. Higher income was associated with significantly higher RBC and serum folate concentrations (Table 5). Serum folic acid concentration was 40% higher in supplement users compared with non-users and was higher in younger children (1.5–10 y) compared with adults. Participants in the white ethnic group had higher folic acid concentrations compared with the mixed, Asian, and “Other” ethnic groups (Supplemental Table 7).

**TABLE 5:** Determinants of red blood cell (RBC) and serum folate concentration in NDNS RP 2008 – 2019^1^

	RBC folate			Serum folate		
Variable	*n*	% difference from reference group	*P*	*n*	% difference from reference group	*P*
Age (y)						
1.5–3	122	29	<0.0001	118	103	<0.0001
4–10	557	11	<0.0001	569	53	<0.0001
11–18	1053	-13	<0.0001	1106	-7	<0.0015
19–64	2856	Ref	Ref	3104	Ref	Ref
65+	807	10	<0.0001	891	17	<0.0001
Sex						
Male	2443	Ref	Ref	2615	Ref	Ref
Female	2952	-4	0.005	3173	5	0.027
BMI category^2^						
Normal & underweight	2383	Ref	Ref	2499	Ref	Ref
Overweight	1559	1	0.62	1704	-3	0.16
Obese	1221	5	0.04	1341	-7	0.014
Country						
England	3434	Ref	Ref	3738	Ref	Ref
Scotland	836	-4	0.19	861	-3	0.45
Wales	686	1	0.59	743	-1	0.66
Northern Ireland	439	-4	0.10	446	-7	0.04
Ethnic group						
White	4927	Ref	Ref	5291	Ref	Ref
Mixed	85	-6	0.15	96	-10	0.07
Black	92	-23	<0.0001	98	-16	0.01
Asian	227	-3	0.36	231	-9	0.13
Other	63	-6	0.37	71	-15	0.06
Smoking						
Nonsmoker	4422	Ref	Ref	4744	Ref	Ref
Smoker	925	-14	<0.0001	990	-17	<0.0001
Supplement use						
No	3946	Ref	Ref	4203	Ref	Ref
Yes	1449	19	<0.0001	1585	32	<0.0001
Equivalized income^3^						
Low	1506	Ref	Ref	1623	Ref	Ref
Mid	1515	1	0.62	1591	8	0.006
High	1734	5	0.035	1872	14	<0.0001

Abbreviations: NDNS RP, National Diet and Nutrition Survey Rolling Program; RBC, red blood cell; Ref, reference group.

1 % difference from the reference group was determined in a multiple linear regression that included all participants with the outcome measure of RBC folate or serum folate. The models included all variables listed in the table and NDNS RP survey year as integer variables. Natural log values of the outcome measures were included and ratios of geometric means between groups were used to calculate the percentage difference between categorical variables.

2 BMI groups were combined into normal and underweight, overweight and obese so that adult and children categories could be combined in a single model. See the Methods section of the main text for further details.

3 Equivalized income is categorized as household income adjusted for different demands on resources considering the household size and composition. See the Methods section of the main text for further details.

Where estimates could be calculated, the prevalence of RBC folate concentration <305 nmol/L and serum folate concentration <13 nmol/L were generally consistent with the patterns observed for group differences in concentration data. The exceptions were with RBC folate deficiency, where there was no difference in older adults or the obese and no difference in the prevalence of serum folate deficiency by ethnicity (Supplemental Table 8). When regression analysis was restricted to only FRA (aged 16–49), results were similar for all participants (Supplemental Table 9). Estimates could not be calculated for all determinants due to an insufficient number of participants within one or more categories. Consequently, the model was not fitted for these determinants, resulting in gaps in the tables. For example, there were insufficient nonwhite participants with RBC folate deficiency; hence, the ethnicity determinant could not be included in the regression analysis.

## Discussion

These nationally representative population data from the United Kingdom NDNS RP show a statistically significant decrease in folate status between 2008 and 2019. The same pattern was observed whether folate status was assessed by RBC folate or serum folate concentration or with established cut-offs for folate deficiency. Deficiency was present in all age groups and was more prevalent in older children (11–18 y), where 17% had an RBC folate concentration <305 nmol/L. Nine out of 10 FRA (16–49 y) had RBC folate concentration less than the cut-off associated with increased risk of NTD (<748 nmol/L).

Notwithstanding concerns over differences in assay and application of cut-offs, as well as differences in the stated age range of children, a recent analysis of nationally representative data from 24 countries, including from United Kingdom NDNS, suggested the prevalence of folate deficiency in United Kingdom children (18 to 48 mo) is low compared with other countries [31]. Stevens et al. [31] reported that in FRA, the United Kingdom had the 12th (of 16) lowest prevalence of folate deficiency, based on a cut-off for megaloblastic anemia. However, in comparison to other high-income countries, Rogers et al. [11] showed that the prevalence of folate deficiency is relatively high in FRA in the United Kingdom, and additionally, our data show that almost 9 out of 10 females have RBC folate concentrations that are associated with an increased risk of NTD. More specifically, published data from the US National Health and Examination Survey (NHANES) revealed that in the period prior to the intriduciton of folic acid fortification (1988-1994), 59% of FRA (12–49 y) had an RBC folate concentration <748 nmol/L, (1988–1994) [32], a figure notably lower than most recently observed in the United Kingdom (89%). Similarly, serum and RBC folate concentrtions in the United Kingdom are closer to median concentrations in the USA prefortification than to recent concentrations that have approximately doubled since the introduction of mandatory fortification [32]. Comparison of NDNS data with other population datasets is complicated because of the use of different assays. The effect of different assays on cut-offs is well-documented [28], and assay-adjusted cut-offs have been used to compare between countries [31]. Attempts have also been made to harmonize previously published folate data to map risk of folate insufficiency [11]. It is established that there is a general lack of population-representative data [11,31,33]. In addition, countries that tend to have the most reliable folate status data introduced folic acid fortification a number of years or decades previously, in contrast to the United Kingdom population with no mandatory fortification.

Folate status was generally lowest in young people aged 11 to 18 y. For females in this age group, this is of particular concern in relation to risk of NTD and teenage pregnancy, although pregnancies in this age group have declined in recent years [34]. The reasons for low folate status in this age group require further study but may be related to diet and/or increased physiologic requirements [1,35]. NDNS RP data reveal that the food groups “cereals and cereal products” and “vegetables and potatoes” contribute the highest proportion of dietary folate [36]. Data also show that compared with older age groups, the 11–18-y-olds had the lowest consumption of fruits and vegetables, consuming on average 2.9 portions per day and with only 12% meeting the 5-a-day recommendation [37].

Examination of the determinants of folate concentration revealed a negative effect of black ethnic group and smoking and a positive effect of supplementation use and higher income. Measured objectively using serum cotinine as a marker of smoking status, Pfeiffer et al. [38] also showed a strong negative effect of smoking on folate status. As expected, supplement users had 19% and 32% higher RBC and serum folate concentrations, respectively, and this was observed despite the lack of granularity on supplement type, dose, and frequency from the single, broad question on supplement usage. The association with income has also been observed previously [39]. Whereas, as already discussed, young people aged 11 to 18 had lower folate status than the adult reference group (19–64 y), younger children and older adults had significantly higher concentrations of both RBC and serum folate. A U-shaped relationship between folate status and age was previously described for NHANES data, both pre- and postfortification [38,39]. BMI category and sex had a smaller but still significant association with folate status, although their direction was not consistent between RBC and serum folate. Similarly, in NHANES, albeit postintroduction of fortification and unadjusted for other factors, a negative association was found between BMI and serum folate and a positive association between BMI and RBC folate [38]. We observed higher serum folate levels in females but lower RBC folate levels compared with males. In NHANES, prior to fortification, females had significantly higher serum folate than males, while there was no difference for RBC folate after correction for other factors such as race and age [39].

Unmetabolized folic acid in serum is derived exclusively from folic acid present in the diet from fortified foods or supplements. Measurement and monitoring of serum folic acid concentrations is of particular interest in relation to mandatory fortification and folate supplementation due to concerns over potential but unproven adverse effects [40,41]. Geometric mean folic acid concentrations observed in United Kingdom adults (0.11–0.39 nmol/L over different NDNS years) were comparable to data from the United States prior to the introduction of mandatory folic acid fortification where the median value was 0.25 nmol/L [42].

Although cross-sectional in design, the NDNS RP and the data presented in this report have a number of strengths, including their representativeness, continuity of data collection and methods, and robust laboratory analysis using gold-standard methods with well-established cut-offs for deficiency. RBC folate by MBA is the established marker of folate status [3,29], and LC-MS/MS provides the most accurate and detailed assessment of serum folate vitamer concentration. As a result, these data provide valuable information on folate deficiency in the United Kingdom and contribute to global estimates. In addition, these data provide information on United Kingdom folate status prior to the announcement and implementation of the mandatory fortification of non-whole wheat flour with folic acid and provide a robust baseline to model and measure the impact of the expected future fortification. However, it should also be recognized that for some age and sex groups, the number of participants was relatively small, with the consequence that descriptive statistics could not be reported for all of the groups for every survey year, particularly for young children. Although the data show that a high proportion of FRA have RBC folate concentration below the cut-off for increased risk of NTD, pregnant or lactating females were excluded from NDNS RP 2008-2019. Although the majority of participants fasted, a single serum folate measurement may not be an accurate measure of an individual’s folate status due to the potential impact of recent folate intake [3,43]. A further limitation is the use of ambient postage for blood samples used to measure RBC folate as per the NDNS RP protocol in Years 1–11. The extent to which delayed processing of WB causes degradation of RBC folate is not certain, with some reports claiming stability up to 24 h [44] and 72 h [45], whereas other studies have reported 10% and 20% loss of RBC folate in blood stored at room temperature for 1 or 2 d [46,47]. Consequently, there is a risk that RBC folate deficiency may be marginally overestimated in the NDNS RP data. However, with the consistency of blood sample collection, processing, and analytical procedures across all survey years reported here, and monitoring and exclusion of samples > 2 d in the post in later years, risk of RBC folate degradation in samples is unlikely to affect conclusions of the trend analysis.

In conclusion, these data reveal a decline in population folate status in the United Kingdom between 2008 and 2019, assessed with both RBC and serum folate. The prevalence of folate deficiency has increased across all age and sex groups. In FRA, in particular, 89% of females had folate concentration less than <748 nmol/L, the cut-off associated with an increased risk of NTD, in 2019. These data provide valuable information on folate status in a population not currently exposed to mandatory folic acid fortification and contribute to global estimates of folate deficiency. Such information is vital to develop and evaluate the expected folic acid fortification program in the United Kingdom following the decision to legislate mandatory fortification of non-whole wheat flour.

### Author contributions

All authors contributed to conception and implementation of the work included in this manuscript. SRM performed serum folate analysis from NDNS Year 7 onwards. DC performed statistical analysis. KSJ drafted the manuscript. All authors have read and approved the final manuscript.

### Conflict of interest

The authors report no conflicts of interest.

### Funding

The United Kingdom National Diet and Nutrition Survey is funded by United Kingdom government: Office for Health Improvement and Disparities (Department of Health and Social Care) and the Food Standards Agency. The survey is currently delivered by NatCen Social Research and the MRC Epidemiology Unit at the University of Cambridge. For the purpose of Open Access, the author has applied a Creative Commons Attribution (CC BY) license to any Author Accepted Manuscript version arising.

This research was also supported by the National Institute for Health and Care Research (NIHR) Cambridge Biomedical Research Centre (NIHR203312). The views expressed are those of the authors and not necessarily those of the NIHR or the Department of Health and Social Care

### Data availability

Data described in the manuscript are available from the United Kingdom Data Service (https://ukdataservice.ac.uk/; https://doi.org/10.5255/UKDA-SN-6533-19).
