# Supplementary material for: National Diet and Nutrition Survey data reveal a decline in folate status in the United Kingdom population between 2008 and 2019
Source: Am J Clin Nutr. 2023 Oct 14;118(6):1182–91. doi: 10.1016/j.ajcnut.2023.10.006 (PMC10739772; doi:10.1016/j.ajcnut.2023.10.006)
Supplement: Multimedia component.1 [file mmc1.docx]

**National Diet and Nutrition Survey data reveal a decline in folate status in the United Kingdom population between 2008 and 2019**

**Kerry S. Jones^1^, David Collins^2^, Sarah R. Meadows^1^, Albert Koulman^1^ and Polly Page^2^**

^1^ Nutritional Biomarker Laboratory, MRC Epidemiology Unit, University of Cambridge, Cambridge, UK

^2^ Nutrition Measurement Platform, MRC Epidemiology Unit, University of Cambridge, Cambridge, UK

**Supplemental Material**

**Supplemental Figure 1.** Comparison of total serum folate (A) and 5-methytetrahydrofolate (B) results for 250 NDNS RP Year 4 (2011/2012) samples between NDNS central laboratory (MRC Elsie Widdowson Laboratory (EWL) and Centers for Disease Control and Prevention (CDC) as assessed during change in analytical laboratory for serum folate. Open symbols are individuals data points and solid line the regression line. Dashed line is the line of identity (x=y).

**Supplemental Table 1.** Results of routine analysis of NIST Standard Reference Material (SRM) 1950^1^ during NDNS RP Years 7 – 11 (2014 - 2019) at the NDNS central laboratory^2^

|  | 5-methyltetrahydrofolate (5-MTHF) | | | | | Folic acid | | | | |
| --- | --- | --- | --- | --- | --- | --- | --- | --- | --- | --- |
|  | Y7 | Y8 | Y9 | Y10 | Y11 | Y7 | Y8 | Y9 | Y10 | Y11 |
| Target (nmol/L) | 26.91 ± 0.70 | | | | | 3.42 ± 1.02 | | | | |
| Mean (nmol/L) | 27.9 | 28.9 | 28.5 | 28.4 | 28.3 | 3.1 | 3.1 | 3.2 | 3.2 | 3.1 |
| SD (nmol/L) | 0.8 | 1.3 | 1.3 | 2.0 | 0.4 | 0.1 | 0.1 | 0.2 | 0.2 | 0.1 |
| %CV | 3.0 | 4.6 | 4.5 | 7.1 | 1.3 | 2.4 | 2.1 | 5.8 | 4.9 | 1.8 |
| n | 7 | 8 | 10 | 9 | 6 | 7 | 8 | 10 | 9 | 6 |

^1^ NIST Standard Reference Material 1950 (https://shop.nist.gov/ccrz__ProductDetails?sku=1950&cclcl=en_US)
^2^ Data were not available for Years 1 – 6 (2008 – 2014)

**Supplemental Figure 2.** Flow diagram of recruitment and response rates for the UK National Diet and Nutrition Survey Rolling Programme Years 1 – 11, 2008 – 2019.^1^

Households invited
n 52,039

Eligible households
n 23,484

Ineligible households^2^
n 28,555

Selected households
n 21,481

Household refused
n 2,003

Productive households^3^
n 13,114

Unproductive households
n 8,367

Productive individuals
n 15,655

RBC and serum folate^4^
n 5,095

Blood sample^4^
n 6,195

Serum folate only^4^
n 300

RBC folate only^4^
n 693

Abbreviations: RBC, red blood cell.

^1^ For further information see Venables et al, 2022 “Data Resource Profile: United Kingdom National Diet and Nutrition Survey Rolling Programme (2008–19)”, International Journal of Epidemiology, Volume 51, Issue 4, Pages e143–e155
^2^ Mainly due to households selected for child boost containing no children. Also ineligible due to being vacant or derelict properties, or institutions
^3^ Productive household defined by completion of 3 or 4 day diet diary
^4^ Multiple blood tubes were collected from consented participants for blood analytes in addition to folate. Differences in numbers reflects the availability of sufficient whole blood or serum to perform folate analyses

**Supplemental Table 2**. Count of red blood cell (RBC) folate concentrations with surrogate values for haematocrit or serum folate, NDNS RP 2008 - 2019^1^

|  | NDNS RP Years | | | | |
| --- | --- | --- | --- | --- | --- |
|  | 1 – 2 | 3 – 4 | 5 – 6 | 7 – 8 | 9 – 11 |
| RBC folate | 1054 | 1345 | 918 | 987 | 1091 |
| With surrogate haematocrit | 23 | 9 | 36 | 54 | 18 |
| With surrogate serum folate | 70 | 79 | 44 | 37 | 37 |

^1^ NDNS RP Years are: Years 1 – 2, 2008/09 – 2009/10); Years 3 – 4, 2010/11 – 2011/2012; Years 5 – 6, 2012/13 – 2013/14; Years 7 – 8, 2014/15 – 2015/16; Years 9 – 11, 2016/17 – 2018/19).

**Supplemental Table 3**. Sample sizes by age and sex groups in NDNS RP 2008 - 2019^1^

|  | | RBC folate, n | | | | | Serum folate, n | | | | |
| --- | --- | --- | --- | --- | --- | --- | --- | --- | --- | --- | --- |
|  | | NDNS RP Years | | | | | | | | | |
| Age | Sex | 1 – 2 | 3 – 4 | 5 – 6 | 7 – 8 | 9 – 11 | 1 – 2 | 3 – 4 | 5 – 6 | 7 – 8 | 9 – 11 |
| 1.5 – 3 y | All | 16 | 17 | 35 | 30 | 24 | 25 | 11 | 30 | 25 | 27 |
| 4 – 10 y | All | 92 | 133 | 93 | 111 | 128 | 103 | 125 | 97 | 102 | 142 |
|  | Male | 44 | 73 | 49 | 63 | 72 | 48 | 75 | 51 | 59 | 81 |
|  | Female | 48 | 60 | 44 | 48 | 56 | 55 | 50 | 46 | 43 | 61 |
| 11 – 18 y | All | 238 | 280 | 163 | 179 | 193 | 254 | 267 | 178 | 177 | 230 |
|  | Male | 120 | 145 | 79 | 93 | 116 | 133 | 137 | 92 | 95 | 130 |
|  | Female | 118 | 135 | 84 | 86 | 77 | 121 | 130 | 86 | 82 | 100 |
| 19 – 64 y | All | 557 | 736 | 479 | 513 | 571 | 594 | 724 | 534 | 525 | 727 |
|  | Male | 236 | 305 | 187 | 220 | 241 | 250 | 295 | 209 | 225 | 303 |
|  | Female | 321 | 431 | 292 | 293 | 330 | 344 | 429 | 325 | 300 | 424 |
| 65+ y | All | 151 | 179 | 148 | 154 | 175 | 170 | 174 | 173 | 153 | 221 |
|  | Male | 63 | 75 | 61 | 63 | 80 | 71 | 72 | 71 | 63 | 94 |
|  | Female | 88 | 104 | 87 | 91 | 95 | 99 | 102 | 102 | 90 | 127 |
| 16 – 49 y | FRA | 250 | 350 | 235 | 208 | 241 | 273 | 343 | 260 | 213 | 302 |

^1^ Sample sizes are unweighted and are actual number of participants per category

Abbreviations: FRA, females of reproductive age; NDNS RP, National Diet and Nutrition Survey Rolling Programme; RBC folate, red blood cell folate

NDNS RP Years are: Years 1 – 2, 2008/09 – 2009/10); Years 3 – 4, 2010/11 – 2011/2012; Years 5 – 6, 2012/13 – 2013/14; Years 7 – 8, 2014/15 – 2015/16; Years 9 – 11, 2016/17 – 2018/19)

**Supplemental Table 4**. General participant characteristics for all NDNS RP 2008 - 2019^1^ participants with valid blood sample collection^1,2^

|  | Age | | | | |
| --- | --- | --- | --- | --- | --- |
|  | 1.5 – 3 y | 4 – 10 y | 11 – 18 y | 19 – 64 y | 65+ y |
| n | 157 | 658 | 1188 | 3235 | 934 |
| Sex (%) |  |  |  |  |  |
| Male | 48 | 55 | 53 | 41 | 42 |
| Female | 52 | 45 | 47 | 59 | 58 |
| BMI-category (%)^3^ |  |  |  |  |  |
| Normal & underweight | 46 | 71 | 67 | 35 | 26 |
| Overweight | 16 | 14 | 13 | 35 | 39 |
| Obese | 12 | 14 | 18 | 26 | 27 |
| Ethnic group (%) |  |  |  |  |  |
| White | 78 | 83 | 90 | 92 | 98 |
| Mixed | 4 | 5 | 3 | 1 | 1 |
| Black | 4 | 3 | 1 | 2 | 1 |
| Asian | 7 | 8 | 5 | 4 | 1 |
| Other | 7 | 1 | 1 | 1 | 0 |
| Smoking (%) |  |  |  |  |  |
| Non-smoker | 100 | 97 | 88 | 75 | 87 |
| Smoker | 0 | 0 | 10 | 25 | 13 |
| Supplement use (%) |  |  |  |  |  |
| No | 72 | 77 | 85 | 71 | 61 |
| Yes | 28 | 23 | 15 | 29 | 39 |
| Equivalised income (%)^4^ |  |  |  |  |  |
| Low | 31 | 29 | 33 | 25 | 32 |
| Mid | 26 | 29 | 29 | 27 | 29 |
| High | 34 | 33 | 26 | 37 | 21 |

^1^ Participant characteristics are generated from all participants who provided a blood sample. Not all participants listed in this table had a result for serum or RBC folate. Characteristics were self-reported except for height and weight used to calculate BMI
^2^ Percentages are rounded and in addition may not add up to 100% due to missing data points and the denominator being the total sample size for each age category
^3^ BMI categories were combined into normal and underweight, overweight and obese so that adult and children categories could be combined in a single model. For further details, see the Methods section of the main text
^4^ Equivalised income is categorised household income adjusted for different demands on resources considering the household size and composition. For further details, see the Methods section of the main text

Abbreviations: BMI, body mass index; NDNS RP, National Diet and Nutrition Survey Rolling Programme

**Supplemental Table 5**. Serum folate concentrations (excluding MeFox) by age and sex groups in NDNS RP 2008 - 2019^1^

|  | | Serum folate, nmol/L (geometric mean (2.5^th^, 97.5^th^ percentiles)) ^2^ | | | | |
| --- | --- | --- | --- | --- | --- | --- |
|  | | NDNS RP years | | | | |
| Age | Sex | 1 – 2 | 3 – 4 | 5 – 6 | 7 – 8 | 9 – 11 |
| 1.5 – 3 y | All | - | - | 30.7 (9.4, 86.9) | - | - |
| 4 – 10 y | All | 25.4 (11.0, 65.0) | 24.8 (7.8, 68.5) | 24.8 (10.7, 56.0) | 21.0 (8.3, 52.6) | 20.1 (6.5, 45.7) |
|  | Male | 30.2 (9.9, 79.1) | 24.7 (7.6, 66.8) | 27.7 (13.1, 54.7) | 20.4 (9.2, 52.4) | 20.9 (5.9, 49.2) |
|  | Female | 22.7 (10.5, 43.5) | 25.0 (6.8, 75.5) | 22.1 (9.5, 51.9) | 21.7 (7.5, 49.2) | 19.2 (6.7, 39.7) |
| 11 – 18 y | All | 15.1 (5.7, 39.8) | 14.2 (4.9, 45.1) | 13.7 (4.8, 38.4) | 10.5 (3.9, 28.0) | 12.1 (5.1, 33.0) |
|  | Male | 14.9 (5.4, 38.1) | 14.8 (4.9, 58.1) | 14.0 (4.4, 37.6) | 11.1 (4.4, 22.7) | 12.1 (4.4, 33.5) |
|  | Female | 15.2 (6.2, 39.8) | 13.6 (5.1, 32.1) | 13.4 (5.4, 38.2) | 9.9 (3.0, 29.6) | 12.1 (5.1, 29.1) |
| 19 – 64 y | All | 16.8 (6.5, 48.0) | 15.4 (5.1, 54.5) | 14.6 (4.7, 53.3) | 13.1 (4.7, 43.1) | 13.0 (4.3, 55.7) |
|  | Male | 16.3 (6.2, 45.7) | 14.1 (4.4, 38.1) | 13.9 (4.7, 53.2) | 12.6 (5.1, 32.6) | 12.0 (4.3, 40.8) |
|  | Female | 17.2 (6.5, 47.9) | 16.8 (5.4, 71.7) | 15.3 (4.9, 53.8) | 13.6 (4.4, 46.7) | 13.9 (4.4, 63.0) |
| 65+ y | All | 19.2 (5.6, 63.7) | 20.2 (7.4, 66.8) | 16.5 (5.2, 52.4) | 16.8 (4.8, 69.8) | 16.1 (4.7, 64.8) |
|  | Male | 17.5 (5.1, 59.5) | 18.1 (7.5, 52.8) | 16.6 (5.2, 50.9) | 13.6 (4.5, 39.0) | 16.4 (4.5, 49.9) |
|  | Female | 20.4 (5.6, 66.3) | 22.1 (7.0, 72.0) | 16.3 (5.0, 48.2) | 19.9 (4.5, 71.0) | 16.0 (5.1, 65.9) |
| 16 – 49 y | FRA | 16.3 (6.5, 44.1) | 15.9 (6.1, 63.4) | 13.7 (4.2, 41.3) | 11.6 (4.4, 42.7) | 13.1 (4.3, 62.3) |

^1^ Where the group size is less than 30 participants the results are not included
^2^ Serum folate concentration is sum of five folate vitamer concentrations [5-methyltetrahydrofolate (5-MTHF), folic acid, tetrahydrofolate (THF), 5-formyltetrahydrofolate (5-FTHF) and 5,10 methenyltetrahydrofolate (CH+THF). Sum excludes pyrazino-s-triazine derivative of 4α-hydroxy-5-methyltetrahydrofolate (MeFox)]

Abbreviations: FRA, females of reproductive age; NDNS RP, National Diet and Nutrition Survey Rolling Programme.

NDNS RP Years are: Years 1 – 2, 2008/09 – 2009/10); Years 3 – 4, 2010/11 – 2011/2012; Years 5 – 6, 2012/13 – 2013/14; Years 7 – 8, 2014/15 – 2015/16; Years 9 – 11, 2016/17 – 2018/19)

**Supplemental Table 6**. Per year change in measure calculated from time trend plots, in NDNS RP 2008 - 2019^1^

| Age | Sex | RBC folate concentration, % | RBC folate <305 nmol/L | Serum folate concentration, % | Serum folate <7 nmol/L | Serum folate <13 nmol/L |
| --- | --- | --- | --- | --- | --- | --- |
|  |  | Change per year^1^ | | | | |
| 1.5 – 3 y | All | -3 (-5, -1) | - | -3 (-6, 1) | - | 1 |
| 4 – 10 y | All | -3 (-4, -2) | - | -3 (-4, -2) | - | 1 (1, 2) |
|  | Male | -3 (-4, -2) | - | -4 (-6, -2) | - | 1 (0.1, 2) |
|  | Female | -3 (-4, -1) | - | -2 (-4, -0.3) | - | - |
| 11 – 18 y | All | -3 (-4, -2) | 2 (1, 2) | -3 (-4, -2) | 1 (0.1, 1) | 2 (1, 3) |
|  | Male | -3 (-4, -2) | 2 (1, 2) | -3 (-4, -1) | 1 (-0.1, 1) | 2 (-0.1, 3) |
|  | Female | -3 (-4, -2) | 1 (0.1, 3) | -3 (-4, -1) | 1 (0.03, 1) | 2 (1, 4) |
| 19 – 64 y | All | -3 (-4, -2) | 1 (0.5, 1) | -3 (-4, -2) | 1 (0.5, 0.1) | 3 (2, 3) |
|  | Male | -2 (-3, -1) | 1 (0.1, 1) | -3 (-4, -3) | 1, (0.1, 1) | 3 (2, 4) |
|  | Female | -3 (-4, -2) | 1 (0.5, 1) | -2 (-4, -1) | 1 (0.7, 1) | 2 (1, 3) |
| 65+ y | All | -3 (-4, -1) | 1 (0.5, 2) | -2 (-4, -0.2) | 1 (0.1, 1) | 1 (-0.5, 2) |
|  | Male | -3 (-5, -1) | - | -2 (-4, -0.1) | 1 (0.5, 1) | 0.1 (2, 2) |
|  | Female | -3 (-4, -1) | 1 (-0.1, 2) | -2 (-4, -0.8) | 1 (-0.2, 1) | 1 (-0.5, 3) |
| 16 – 49 y | FRA | -3 (-4, -2) | 1 (1, 2) | -3 (-4, -1) | - | 3 (2, 4) |

^1^ For red blood cell (RBC) and serum folate concentration, the change per year is average percent change per year; for cut-offs the values are percentage point change per year. If 95% CI does not include zero then the change is significant (P<0.05). Cells with a dash indicate that due to the low numbers it was not possible to calculate the change per year

Abbreviations: FRA, females of reproductive age

**Supplemental Table 7**. Determinants of folic acid concentration in NDNS RP 2008 - 2019^1^

|  | Folic acid | | |
| --- | --- | --- | --- |
| Variable | n | % difference from reference group | P-value |
| Age (years) |  |  |  |
| 1.5 – 3 | 120 | 118 | <0.0001 |
| 4 – 10 | 576 | 25 | <0.0001 |
| 11 – 18 | 1115 | 1 | 0.81 |
| 19 – 64 | 3116 | Ref | Ref |
| 65+ | 893 | 9 | 0.13 |
| Sex |  |  |  |
| Male | 2633 | Ref | Ref |
| Female | 3187 | 7 | 0.065 |
| BMI category^2^ |  |  |  |
| Normal & underweight | 2514 | Ref | Ref |
| Overweight | 1709 | -5 | 0.27 |
| Obese | 1352 | -3 | 0.56 |
| Country |  |  |  |
| England | 3755 | Ref | Ref |
| Scotland | 875 | -7 | 0.25 |
| Wales | 743 | -8 | 0.22 |
| Northern Ireland | 447 | 2 | 0.78 |
| Ethnic group |  |  |  |
| White | 5319 | Ref | Ref |
| Mixed | 97 | -28 | 0.04 |
| Black | 99 | 6 | 0.57 |
| Asian | 233 | -26 | 0.0002 |
| Other | 71 | -36 | 0.0002 |
| Smoking |  |  |  |
| Non-smoker | 4769 | Ref | Ref |
| Smoker | 996 | -1 | 0.85 |
| Supplement use |  |  |  |
| No | 4230 | Ref | Ref |
| Yes | 1590 | 40 | <0.0001 |
| Equivalised income^3^ |  |  |  |
| Low | 1635 | Ref | Ref |
| Mid | 1598 | -2 | 0.75 |
| High | 1882 | 2 | 0.63 |

^1^ % difference from the reference group was determined in a multiple linear regression that included all participants with the outcome measure of folic acid concentration. The models included all variables listed in the table and NDNS RP survey year as integer variables. Natural log values of the outcome measures were included and ratios of geometric means between groups were used to calculate the percentage difference between categorical variables
^2^ BMI categories were combined into normal and underweight, overweight and obese so that adult and children categories could be combined in a single model. For further details, see the Methods section of the main text
^3^ Equivalised income is categorised household income adjusted for different demands on resources considering the household size and composition. For further details, see the Methods section of the main text

Abbreviations: BMI, body mass index; NDNS RP, National Diet and Nutrition Survey Rolling Programme; RBC, red blood cell; Ref, reference group

**Supplementary Table 8**. Determinants of the prevalence of folate deficiency based on red blood cell (RBC) and serum folate concentrations in NDNS RP 2008 - 2019^1^

|  | RBC folate % <305 nmol/L | | | Serum folate % <13 nmol/L | | |
| --- | --- | --- | --- | --- | --- | --- |
| Variable | n | % difference from reference group | P-value | n | % difference from reference group | P-value |
| Age (years) |  |  |  |  |  |  |
| 11 – 18 | 1053 | 6.2 | <0.0001 | 1106 | 6.8 | 0.006 |
| 19 – 64 | 2856 | Ref | Ref | 3104 | Ref | Ref |
| 65+ | 807 | 0.2 | 0.89 | 891 | -10.1 | 0.0002 |
| Sex |  |  |  |  |  |  |
| Male | - | - | - | 2240 | Ref | Ref |
| Female | - | - | - | 2861 | -2.1 | 0.29 |
| BMI category^2^ |  |  |  |  |  |  |
| Normal & underweight | 1933 | Ref | Ref | 2053 | Ref | Ref |
| Overweight | 1463 | 1.4 | 0.18 | 1601 | 3.5 | 0.14 |
| Obese | 1131 | 0.1 | 0.96 | 1248 | 6.7 | 0.007 |
| Country |  |  |  |  |  |  |
| England | - | - | - | 3244 | Ref | Ref |
| Scotland | - | - | - | 770 | 5.4 | 0.11 |
| Wales | - | - | - | 652 | 1.8 | 0.55 |
| Northern Ireland | - | - | - | 435 | 4.5 | 0.20 |
| Ethnic group |  |  |  |  |  |  |
| White | - | - | - | 4724 | Ref | Ref |
| Mixed | - | - | - | 67 | 10.9 | 0.23 |
| Black | - | - | - | 76 | 8.5 | 0.30 |
| Asian | - | - | - | 178 | -0.7 | 0.89 |
| Other | - | - | - | 55 | 7 | 0.40 |
| Smoking |  |  |  |  |  |  |
| Non-smoker | 3761 | Ref | Ref | 4075 | Ref | Ref |
| Smoker | 924 | 5.6 | 0.0002 | 989 | -16.4 | <0.0001 |
| Supplement use |  |  |  |  |  |  |
| No | - | - | - | - | - | - |
| Yes | - | - | - | - | - | - |
| Equivalised income^3^ |  |  |  |  |  |  |
| Low | 1314 | Ref | Ref | 1419 | Ref | Ref |
| Mid | 1313 | -4.7 | 0.004 | 1397 | -8.8 | 0.0006 |
| High | 1508 | -7.4 | <0.0001 | 1642 | -12.1 | <0.0001 |

^1^ % difference from the reference group was determined in a multiple linear regression that included all participants with the outcome measure of RBC folate or serum folate. The models included all variables listed in the table and NDNS RP survey year as integer variables. Natural log values of the outcome measures were included and ratios of geometric means between groups were used to calculate the percentage difference between categorical variables. Gaps in the tables result from insufficient participants within one or more categories which meant the model could not be fitted for the given determinant
^2^ BMI categories were combined into normal and underweight, overweight and obese so that adult and children categories could be combined in a single model. For further details, see the Methods section of the main text
^3^ Equivalised income is categorised household income adjusted for different demands on resources considering the household size and composition. For further details, see the Methods section of the main text

Abbreviations: BMI, body mass index; NDNS RP, National Diet and Nutrition Survey Rolling Programme; RBC, red blood cell; Ref, reference group

**Supplemental Table 9**. Determinants of the red blood cell (RBC) folate concentration and prevalence of folate concentration below selected cut-offs in females of reproductive age (FRA) in NDNS RP 2008 - 2019^1^

|  | RBC folate, nmol/L | | | RBC folate, <305 nmol/L | | | RBC folate, <748 nmol/L | | |
| --- | --- | --- | --- | --- | --- | --- | --- | --- | --- |
| Variable | n | % difference from reference group | P-value | n | % difference from reference group | P-value | n | % difference from reference group | P-value |
| BMI category^2^ |  |  |  |  |  |  |  |  |  |
| Normal & underweight | 621 | Ref | Ref | 621 | Ref | Ref | 621 | Ref | Ref |
| Overweight | 337 | -7 | 0.07 | 337 | 6 | 0.04 | 337 | 0.6 | 0.82 |
| Obese | 295 | 5 | 0.20 | 295 | 0.6 | 0.81 | 295 | -4 | 0.26 |
| Country |  |  |  |  |  |  |  |  |  |
| England | 793 | Ref | Ref | 793 | Ref | Ref | - | - | - |
| Scotland | 192 | -8 | 0.15 | 192 | 8 | 0.18 | - | - | - |
| Wales | 159 | 5 | 0.40 | 159 | -0.8 | 0.79 | - | - | - |
| Northern Ireland | 140 | -12 | 0.008 | 140 | 7 | 0.15 | - | - | - |
| Ethnic group |  |  |  |  |  |  |  |  |  |
| White | 1153 | Ref | Ref | - | - | - | - | - | - |
| Mixed | 24 | -13 | 0.11 | - | - | - | - | - | - |
| Black | 25 | -3 | 0.74 | - | - | - | - | - | - |
| Asian | 63 | 6 | 0.34 | - | - | - | - | - | - |
| Other | 19 | -2 | 0.84 | - | - | - | - | - | - |
| Smoking |  |  |  |  |  |  |  |  |  |
| Non-smoker | 972 | Ref | Ref | 972 | Ref | Ref | 972 | Ref | Ref |
| Smoker | 306 | -16 | <0.0001 | 306 | 15.4 | <0.0001 | 306 | 15 | <0.0001 |
| Supplement use |  |  |  |  |  |  |  |  |  |
| No | 923 | Ref | Ref | - | - | - | - | - | - |
| Yes | 361 | 26 | <0.0001 | - | - | - | - | - | - |
| Equivalised income^3^ |  |  |  |  |  |  |  |  |  |
| Low | 395 | Ref | Ref | - | - | - | - | - | - |
| Mid | 343 | 9 | 0.04 | - | - | - | - | - | - |
| High | 403 | 15 | 0.0002 | - | - | - | - | - | - |

^1^ % difference from the reference group in FRA (aged 16 – 49 years) was determined in a multiple linear regression that included all participants with the outcome measure of RBC folate or serum folate. The models included all variables listed in the table and NDNS RP survey year as integer variables. Natural log values of the outcome measures were included and ratios of geometric means between groups were used to calculate the percentage difference between categorical variables. Dashes indicate the variable could not be fitted in the regression model. Gaps in the tables result from insufficient participants within one or more categories which meant the model could not be fitted for the given determinant
^2^ BMI categories were combined into normal and underweight, overweight and obese so that adult and children categories could be combined in a single model. For further details, see the Methods section of the main text
^3^ Equivalised income is categorised household income adjusted for different demands on resources considering the household size and composition. For further details, see the Methods section of the main text

Abbreviations: BMI, body mass index; NDNS RP, National Diet and Nutrition Survey Rolling Programme; RBC, red blood cell; Ref, reference group
